# Predictors of vertebral fractures in patients with severe osteoporosis: a sub-analysis of the Japanese Osteoporosis Intervention Trial-05 (JOINT-05)

**DOI:** 10.1007/s11657-025-01649-7

**Published:** 2025-12-14

**Authors:** Hiroyuki Inose, Shiro Tanaka, Satoshi Mori, Hiroshi Hagino, Satoshi Soen

**Affiliations:** 1 Department of Orthopedic Surgery, Dokkyo Medical University Saitama Medical Center, 2-1-50, Minami-Koshigaya, Koshigaya-Shi, Saitama, 343-8555 Japan; 2 Department of Clinical Biostatistics, Graduate School of Medicine, Kyoto University, Kyoto, Japan; 3 Department of Bone and Joint Surgery, Seirei Hamamatsu General Hospital, Hamamatsu, Shizuoka Japan; 4 Department of Rehabilitation, Sanin Rosai Hospital, Yonago, Tottori Japan; 5 Osteoporosis and Rheumatology Clinic, Soen Orthopaedics, Kobe, Hyogo Japan

**Keywords:** Vertebral fractures, Teriparatide, Alendronate, Risk factor, Triglyceride level, Low back pain

## Abstract

***Summary*:**

Incident vertebral fractures are a major issue in elderly women with severe osteoporosis. Beyond well-established risk factors, osteoporosis treatment given, low serum triglyceride levels, and intensity of low back pain were identified as predictors of new fractures in high-risk elderly women. Clinical strategies need to take these findings into account.

**Purpose:**

Vertebral fractures (VFs) are a major cause of morbidity and economic burden in aging populations. Despite effective osteoporosis treatments, VF incidence continues to increase. This study aimed to identify predictors for incident VFs in elderly women with severe osteoporosis.

**Methods:**

This was a post hoc analysis of data of 778 women aged ≥ 75 years with severe osteoporosis from the JOINT-05 trial. We investigated potential predictors for incident VFs, including the burden of prevalent vertebral fractures (number and Genant grade), prior osteoporosis treatment, lipid profile (e.g., triglycerides), low back pain VAS, physical function (timed-up-and-go, one-leg standing), and nutritional indices (e.g., albumin, selected nutrients from the Food Frequency Questionnaire). Multivariate Poisson regression analysis was used to identify independent predictors.

**Results:**

During the study, 138 participants (17.7%) developed incident VFs. The allocated osteoporosis treatment, the number and grade of pre-existing VFs, prior history of osteoporosis treatment, lower serum triglyceride levels, and the low back pain score were significant predictors of incident VFs. VF incidence was significantly higher in patients with pre-existing VFs, although risk did not increase with the severity of existing VFs.

**Conclusion:**

Beyond established risk factors such as pre-existing fractures, this study identified the allocation of osteoporosis treatment, low serum triglyceride levels, and the intensity of low back pain as independent predictors of new VFs in high-risk elderly women. Thus, clinical strategies to prevent subsequent fractures should include optimizing pharmacological treatment (e.g., sequential therapy with teriparatide), considering lipid status, and implementing effective, multimodal pain management.

## Introduction

Vertebral fractures (VFs) represent a significant and growing public health concern, particularly with the progressive aging of global populations. In the USA, approximately 700,000 new osteoporotic VFs were estimated to occur annually [1]. These fractures are associated with substantial morbidity, including chronic pain, spinal deformity, reduced physical function, and increased mortality [2, 3]. The economic burden is also considerable, with direct medical costs related to osteoporotic fractures, including VFs, estimated to be in the tens of billions of dollars each year in the USA [4]. The total annual expense of providing care for osteoporotic fractures in Medicare beneficiaries has been estimated at 57 billion US dollars in 2018, with an expected increase to over 95 billion US dollars in 2040 [5]. In Japan, vertebral fractures are also highly prevalent, with estimates suggesting roughly 1–4 million cases annually [6, 7]. The combined economic burden of hip and vertebral fractures has been estimated at ~ 27.5 billion JPY, and per-patient acute care costs for fragility fractures remain substantial [8]. Beyond clinical morbidity, VFs pose socioeconomic challenges by increasing healthcare utilization and long-term care needs, while subsequent fractures amplify costs over multiple years for patients and payers [9]. Consequently, the prevention of VFs is not merely a medical imperative, but also a pressing socioeconomic issue.

In recent years, the therapeutic landscape for osteoporosis has evolved significantly. Beyond established anti-resorptive agents such as bisphosphonates and denosumab, potent bone anabolic therapies, including a parathyroid hormone receptor 1 agonist and romosozumab, have become available for clinical use [10]. The efficacy of these treatments in reducing the risk of osteoporotic fractures, including VFs, has been demonstrated in numerous clinical trials and confirmed in real-world clinical practice [11–14]. Furthermore, in recent years, sequential therapy with an initial course of a bone formation-promoting agent followed by an anti-resorptive agent has been recommended for the treatment of osteoporosis in patients at high risk of fracture [11]. Despite these pharmacological advancements, the overall incidence of VFs continues to increase in many aging societies [15, 16]. Established risk factors for osteoporotic fractures include low bone mineral density (BMD), a history of previous fractures, low body mass index (BMI), and heavy alcohol consumption; however, the risk factors for new VFs, particularly in patients with severe osteoporosis, have not yet been fully elucidated [17, 18]. Identifying additional, potentially modifiable risk factors for the occurrence of new VFs is crucial for enhancing prevention strategies and improving patient outcomes. This study aimed to identify predictors associated with the occurrence of VFs in patients with severe osteoporosis.

## Methods

### Study design and ethical considerations

A post hoc analysis focused on identifying predictors of incident VFs was performed using anonymized data from JOINT-05, a Japanese nationwide study that included 1011 patients from 113 institutes between October 2014 and December 2017 [17–19]. The study was conducted in accordance with the Declaration of Helsinki and was approved by the central and onsite institutional review boards. Informed consent was obtained from all participants. We pre-specified 64 baseline variables as candidate predictors (clinical, functional, biochemical, and nutritional domains).

### Participants

A total of 778 women with severe osteoporosis were included in the study cohort. The baseline characteristics of the participants are presented in Table 1. The mean age was 81.5 years, and 69.7% of the participants had pre-existing VFs at baseline (Table 1). The design and primary results of JOINT-05 have been reported previously [19]. The original inclusion criteria for the trial were the following: Japanese women at least 75 years old; diagnosis of primary osteoporosis based on the 2012 revised Japanese Society for Bone and Mineral Research criteria [20]; and a high fracture risk. This high-risk status was defined by one or more of the following: BMD less than 60% of the young adult mean (or < −3.3 SDs); at least two VFs (Th4-L4); a grade 3 pre-existing fracture; or a prior hip fracture.

**Table 1:** Baseline characteristics of 778 postmenopausal women with severe osteoporosis in the analysis population of morphometric vertebral fractures

	Mean or percent	SD
Treatment by Teriparatide → Alendronate sequential therapy	54.9%	
Age (y)	81.5	4.6
Age at menopause (y)	49.4	4.5
Years from menopause	32.1	6.6
Count of pre-existing vertebral fractures	1.8	2.0
Maximum grade of pre-existing vertebral fractures		
Grade 1	9.8%	
Grade 2	17.6%	
Grade 3	42.3%	
History of proximal femoral fractures	14.5%	
Prior treatment	55.3%	
Prior bisphosphonates	30.5%	
BMD at L2-L4 (*T*-score)	−2.321	1.416
Comorbidities		
Hypertension	39.6%	
Diabetes mellitus	8.6%	
Dyslipidemia	16.7%	
Rheumatoid arthritis	0.9%	
Osteoarthritis	0.1%	
Others	29.8%	
MMSE	26.5	4.0
Timed-up-and-go test (seconds)	13.5	9.7
One-leg standing test (seconds)	14.9	23.2
Nursing level		
Support not required	78.9%	
Support required	11.4%	
Level 1	4.1%	
Level 2	3.3%	
Level 3	1.5%	
Level 4 or 5	0.8%	
Height (cm)	146.2	6.3
Weight (kg)	47.7	8.7
BMI (kg/m^2^)	22.3	3.7
Systolic blood pressure (mmHg)	136.3	19.6
Diastolic blood pressure (mmHg)	74.1	13.2
Osteocalcin (ng/mL)	18.5	11.4
PINP (μg/L)	56.2	43.0
TRACP-5b (mU/dL)	479.8	221.9
25(OH)D (ng/mL)	17.5	5.7
Pentosidine (pmol/mL)	34.3	30.2
Corrected pentosidine (pmol/mL∙Cr)	45.2	23.2
HbA1C (%)	5.9	0.5
Total cholesterol (mg/dL)	201.5	36.4
HDL cholesterol (mg/dL)	61.4	16.1
LDL cholesterol (mg/dL)	114.1	31.1
Triglyceride (mg/dL)	117.3	60.5
eGFR (mL/min/1.73 m^2^)	62.6	16.7
Creatinine (mg/dL)	0.7	0.2
Urine creatinine (mg/dL)	75.6	55.7
Albumin (g/dL)	4.1	0.3
Ca (mg/dL)	9.5	0.5
Urine Ca (mg/dL)	11.3	8.5
VAS at rest (0 to 100 points)	21.2	26.4
VAS on motion (0 to 100 points)	33.8	30.9
EQ-5D (utility)	0.729	0.185
Alcohol intake (g)	9.5	37.3
Energy intake (kcal)	1562.9	330.0
Protein intake (g)	71.5	20.1
Fat intake (g)	56.9	15.7
Carbohydrate intake (g)	192.8	44.1
Salt intake (g)	10.1	1.9
Ca intake (mg)	503.1	164.5
Fe intake (mg)	8.3	2.5
Vitamin A intake (μg RE)	1067.1	730.6
Vitamin D intake (μg)	11.3	2.6
Vitamin K intake (μg)	265.7	170.7
Vitamin B1 intake (mg)	0.9	0.2
Vitamin B2 intake (mg)	1.2	0.4
Vitamin B6 intake (mg)	1.1	0.3
Vitamin B12 intake (μg)	5.8	4.0
Folic acid intake (μg)	254.8	99.2
Vitamin C intake (mg)	82.3	29.8
Mg intake (mg)	208.6	51.0
Number of teeth present	11.4	10.2

*SD* standard deviation, *BMD* bone mineral density, *MMSE* Mini-Mental State Examination, *BMI* body mass index, *PINP* procollagen type 1 N-terminal propeptide, *TRACP-5b* tartrate-resistant acid phosphatase-5b, *25(OH)D* 25-hydroxyvitamin D, *HbA1C* hemoglobin A1c, *HDL* high-density lipoprotein, *LDL* low-density lipoprotein, *eGFR* estimated glomerular filtration rate, *Ca* calcium, *VAS* visual analog scale, *EQ-5D* The EuroQol 5 dimensions, *Fe* iron, *Mg* magnesium

Exclusion criteria included secondary osteoporosis from pre-identified causes, other diseases causing bone loss or impacting vertebral strength, history of hypersensitivity, contraindications to study drugs, severe kidney, liver, or heart conditions, hospitalization, or previous use of teriparatide.

### Treatments

In the original JOINT-05 trial, patients were randomized (1:1) to either sequential therapy (56.5 μg subcutaneous teriparatide weekly for 72 weeks, then alendronate for 48 weeks) or continuous alendronate monotherapy for 120 weeks. Alendronate administration included options such as a 5-mg daily oral tablet, a 35-mg weekly oral tablet or jelly, or a 900-μg intravenous infusion every 4 weeks. In addition, daily supplements of 400 IU of native vitamin D were supplied to all participants in both arms for the duration of their treatment [17].

### Baseline measurements

At baseline, demographics, medical history, nursing level status, height, weight, body mass index (BMI), systolic and diastolic blood pressure, and number of teeth present were recorded. Nursing level was classified as “support not required,” “support required” (yō-shien, indicating a condition in which partial assistance is needed), or “nursing care” (yō-kaigo), which is further divided into levels 1 to 5. The nursing care levels reflect the degree of assistance required in daily life or due to cognitive decline, with higher levels indicating a greater need for comprehensive support.

Physical ability was evaluated using the timed-up-and-go (TUG) test and the one-leg standing test (OLST) performed with eyes open. The TUG test involved measuring the total time taken for a patient to rise from a standard chair, walk a distance of 3 m, turn, return to the chair, and sit down again. For the OLST, the duration a patient could maintain a one-legged stance was recorded, with the test ceasing when significant body sway indicated a potential loss of balance, the raised leg touched the floor, or the supporting leg shifted, whichever occurred first. The intensity of back pain was assessed both at rest and during movement using a visual analog scale (VAS) ranging from 0 to 100 points (0 = no pain; 100 = worst imaginable). Because published cut-points vary (e.g., TUG ≥ 13.5 s indicating higher fall risk in meta-analytic reviews [20]; OLST cut-points around < 10 s discussed in recent literature [21]; VAS cutoff points mild < 35, moderate or severe ≥ 35 mm [22]), we analyzed these measures as continuous variables in the primary models. Although longitudinal measurements were obtained in JOINT-05, these variables were treated as baseline predictors of incident vertebral fractures in the present analysis. Cognitive function was assessed using the Mini-Mental State Examination (MMSE). Health-related quality of life was measured using the EQ-5D utility score.

BMD at the lumbar spine was measured at baseline in each institution by dual-energy X-ray absorptiometry. Blood samples were obtained at baseline to measure serum levels of bone turnover markers (osteocalcin, procollagen type I amino-terminal propeptide (PINP), and tartrate-resistant acid phosphatase-5b (TRACP-5b), 25(OH)D, HbA1C, total cholesterol, HDL cholesterol, LDL cholesterol, triglycerides, creatinine, albumin, and calcium). Urine samples were collected at baseline to measure urinary pentosidine, urinary creatinine, and urinary calcium levels. The corrected urinary pentosidine value was calculated using the following formula: [urinary pentosidine (pmol/mL)/urinary creatinine (mg/dL)] × 100.

Nutrient intake was captured by Food Frequency Questionnaire (FFQ) for macro- and micronutrients (e.g., energy, protein, vitamin D, vitamin K, calcium, etc.; see Table 1), noting that dietary vitamin D intake is distinct from serum 25(OH)D levels [21].

### Outcome measures

Thoracic and lumbar vertebrae were imaged bi-directionally at baseline and at 24, 48, 72, and 120 weeks. To identify pre-existing VFs, investigators analyzed anteroposterior and lateral radiographs of the spine (Th4-L4), applying a semiquantitative (SQ) grading method [22]. These initial findings underwent central review by a treatment-blinded evaluator from the fracture assessment committee.

The committee further determined the incidence of VFs (Th4-L4) by comparing baseline and post-treatment radiographs. Two evaluators, blinded to treatment allocation, independently assessed these films using the SQ technique; discrepancies were resolved through a simultaneous review by three evaluators. In this study, an incident VF was defined as the new occurrence of a VF with a Genant’s SQ grade of 1 or higher.

### Statistical analysis

Baseline characteristics and laboratory measurements are reported as means with SD or as percentages. To explore risk factors for VF incidence, 64 variables that were candidates for fracture risk factors among baseline characteristics were first identified based on clinical knowledge and pre-specified in the statistical analysis plan. Second, univariate Poisson regression was then fitted to the associations between these 64 variables and VF incidence. Finally, to select covariates in multivariate‐adjusted Poisson regression models, stepwise variable selection was applied with a cutoff *p*-value ≤ 0.25. The stepwise procedure we used is bidirectional, with a significance level of 0.05 for entry and retention.

In addition to the primary stepwise-selected model, we performed supplementary multivariate analyses using block-wise augmented models to address concerns about the potential omission of clinically relevant variables. Specifically, we constructed three alternative models by adding sets of clinically related variables to the core adjustment set, which consisted of the variables retained in the stepwise selection procedure:

- Physical Function (PF) model, which additionally included timed-up-and-go test and nursing care level;
- Bone Turnover Marker (BTM) model, which additionally included TRACP-5b, urinary pentosidine, and lumbar spine BMD;
- Nutrition/Social (NS) model, which additionally included serum albumin, alcohol intake, hemoglobin A1c (HbA1c), and number of teeth present.

These block-wise models were compared with the core model to evaluate the robustness of predictors of incident vertebral fractures. Because none of the added variables in the BTM or NS models reached statistical significance, an integrated model combining variables across blocks was not pursued. All reported *p*-values are two-tailed, and *p* < 0.05 was considered significant. Missing data were handled by multiple imputation. All statistical analyses were performed by academic statisticians using SAS Version 9.4 (SAS Institute, Cary, NC, USA).

## Results

During the study period, 138 cases (17.7%) and 190 vertebrae developed new VFs. Whether the presence of VFs at baseline affected the incidence of new VFs was investigated first. The incidence of new VFs in patients without pre-existing VFs was 2.0%. In contrast, the incidence was significantly higher in patients with baseline VFs of grade 1 (21%), grade 2 (21%), and grade 3 (15%) (Table 2). However, no clear trend of increasing risk was observed corresponding to the severity grade of the existing VFs.

**Table 2:** Incidence of morphometric vertebral fracture by maximum grade of pre-existing fracture

	Count	Persons	Person-years	Incidence rate
Without pre-existing vertebral fractures (*N* = 236)	9	8	449.4	0.02
Grade 1 (*N *= 76)	29	21	140.2	0.21
Grade 2 (*N *= 137)	56	41	261.8	0.21
Grade 3 (*N *= 329)	96	68	620.3	0.15

Next, factors associated with the occurrence of incident VFs were examined. Univariate analysis (Table 3) indicated that the following factors were significantly associated with new VFs: allocation of osteoporosis treatment (Bisphosphonate monotherapy vs. Teriparatide sequential therapy: RR 1.46, 95% CI 1.08–1.98, *p* = 0.01), age (RR 1.04, 95% CI 1.01–1.07, *p* = 0.02), count of pre-existing vertebral fractures (RR 1.22, 95% CI 1.16–1.29, *p* < 0.01), maximum grade of pre-existing vertebral fractures (e.g., Grade 1 vs. None: RR 10.33, 95% CI 4.89–21.82, *p* < 0.01), lower BMD at L2-L4 (*T*-score) (RR 0.84, 95% CI 0.72–0.98, *p* = 0.03), Nursing level 1 (RR 1.94, 95% CI 1.14–3.30, *p* = 0.01), lower height (RR 0.96, 95% CI 0.94–0.98, *p* < 0.01), lower weight (RR 0.97, 95% CI 0.95–0.99, *p* < 0.01), higher serum TRACP-5b (per 100 mU/dL: RR 1.10, 95% CI 1.04–1.17, *p* < 0.01), higher pentosidine (RR 1.01, 95% CI 1.00–1.01, *p* < 0.01), lower HbA1C (RR 0.71, 95% CI 0.52–0.96, *p* = 0.03), lower total cholesterol (RR 0.99, 95% CI 0.99–1.00, *p* < 0.01), higher HDL cholesterol (RR 1.01, 95% CI 1.00–1.02, *p* = 0.03), lower LDL cholesterol (RR 0.99, 95% CI 0.99–1.00, *p* < 0.01), lower triglycerides (RR 0.99, 95% CI 0.99–1.00, *p* < 0.01), higher urine creatinine (RR 1.00, 95% CI 1.00–1.01, *p* < 0.01), lower albumin (RR 0.60, 95% CI 0.41–0.90, *p* = 0.01), higher VAS at rest (per 10 points: RR 1.13, 95% CI 1.08–1.19, *p* < 0.01), higher VAS on motion (per 10 points: RR 1.09, 95% CI 1.05–1.14, *p* < 0.01), lower EQ-5D (utility) (RR 0.33, 95% CI 0.15–0.71, *p* < 0.01), and alcohol intake (RR 1.00, 95% CI 1.00–1.01, *p* < 0.01).

**Table 3:** Univariable analysis of associations between morphometric vertebral fractures and baseline characteristics

	Rate ratio	95% CI		*p*
Allocation of osteoporosis treatment Bisphosphonate monotherapy (Reference = Teriparatide → Alendronate sequential therapy)	1.46	1.08	1.98	0.01*
Age (y)	1.04	1.01	1.07	0.02*
Age at menopause (y)	1.00	0.97	1.03	0.93
Years from menopause	1.02	1.00	1.04	0.09
Count of pre-existing vertebral fractures	1.22	1.16	1.29	< 0.01*
Maximum grade of pre-existing vertebral fractures				
None	Reference			
Grade 1	10.33	4.89	21.82	< 0.01*
Grade 2	10.68	5.28	21.59	< 0.01*
Grade 3	7.73	3.90	15.31	< 0.01*
History of proximal femoral fractures	0.79	0.50	1.23	0.29
Prior treatment	0.79	0.60	1.05	0.11
Prior bisphosphonates	0.93	0.68	1.27	0.66
BMD at L2-L4 (*T*-score)	0.84	0.72	0.98	0.03*
Comorbidity				
Hypertension	0.99	0.74	1.33	0.97
Diabetes mellitus	0.78	0.45	1.38	0.40
Dyslipidemia	1.23	0.86	1.75	0.25
Rheumatoid arthritis	Not estimable			
Osteoarthritis	3.38	0.47	24.09	0.22
Others	1.21	0.90	1.63	0.21
MMSE	0.98	0.95	1.01	0.25
Timed-up-and-go test (seconds)	1.01	1.00	1.02	0.05
One-leg standing test (seconds)	0.99	0.98	1.00	0.05
Nursing level				
Support not required	Reference			
Support required	1.15	0.75	1.77	0.53
Level 1	1.94	1.14	3.30	0.01*
Level 2	0.35	0.09	1.40	0.14
Level 3	0.80	0.20	3.21	0.75
Level 4 or 5	1.28	0.32	5.15	0.73
Number of teeth present	0.98	0.97	1.00	0.05
Height (cm)	0.96	0.94	0.98	< 0.01*
Weight (kg)	0.97	0.95	0.99	< 0.01*
BMI < 18.5 kg/m^2^	0.89	0.58	1.38	0.61
BMI 18.5–24 kg/m^2^	Reference			
BMI ≥ 25 kg/m^2^	0.87	0.60	1.25	0.44
Systolic blood pressure (mmHg)	1.00	0.99	1.01	0.78
Diastolic blood pressure (mmHg)	1.00	0.98	1.01	0.44
Osteocalcin (ng/mL)	1.00	0.98	1.01	0.50
PINP (μg/L)	1.00	1.00	1.00	0.67
TRACP-5b (per 100 mU/dL)	1.10	1.04	1.17	< 0.01*
25(OH)D (ng/mL)	0.99	0.96	1.01	0.33
Pentosidine (pmol/mL)	1.01	1.00	1.01	< 0.01*
Corrected pentosidine (pmol/mL∙Cr)	1.00	1.00	1.01	0.35
HbA1C (%)	0.71	0.52	0.96	0.03*
Total cholesterol (mg/dL)	0.99	0.99	1.00	< 0.01*
HDL cholesterol (mg/dL)	1.01	1.00	1.02	0.03*
LDL cholesterol (mg/dL)	0.99	0.99	1.00	< 0.01*
Triglyceride (mg/dL)	0.99	0.99	1.00	< 0.01*
eGFR (mL/min/1.73 m^2^)	0.99	0.98	1.00	0.07
Creatinine (mg/dL)	1.47	0.79	2.74	0.23
Urine creatinine (mg/dL)	1.00	1.00	1.01	< 0.01*
Albumin (g/dL)	0.60	0.41	0.90	0.01*
Ca (mg/dL)	1.02	0.74	1.40	0.92
Urine Ca (mg/dL)	1.01	0.99	1.02	0.54
VAS at rest (per 10 points from 0 to 100 points)	1.13	1.08	1.19	< 0.01*
VAS on motion (per 10 points from 0 to 100 points)	1.09	1.05	1.14	< 0.01*
EQ-5D (utility)	0.33	0.15	0.71	< 0.01*
Alcohol intake	1.00	1.00	1.01	< 0.01*
Energy intake	1.00	1.00	1.00	0.98
Protein intake	1.00	0.99	1.00	0.35
Fat intake	1.00	0.99	1.00	0.34
Carbohydrate intake	1.00	1.00	1.00	0.66
Salt intake	1.05	0.98	1.13	0.16
Ca intake	1.00	1.00	1.00	0.69
Fe intake	1.00	0.94	1.06	1.00
Vitamin A intake	1.00	1.00	1.00	0.32
Vitamin D intake	1.01	0.95	1.06	0.82
Vitamin K intake	1.00	1.00	1.00	0.80
Vitamin B1 intake	0.87	0.47	1.61	0.65
Vitamin B2 intake	0.99	0.71	1.37	0.94
Vitamin B6 intake	0.88	0.54	1.42	0.59
Vitamin B12 intake	0.98	0.95	1.02	0.41
Folic acid intake	1.00	1.00	1.00	0.80
Vitamin C intake	1.00	0.99	1.00	0.67
Mg intake	1.00	1.00	1.00	0.74

*p < 0.05*CI* confidence interval, *BMD* bone mineral density, *MMSE* Mini-Mental State Examination, *BMI* body mass index, *PINP* procollagen type 1 N-terminal propeptide, *TRACP-5b* tartrate-resistant acid phosphatase-5b, *25(OH)D* 25-hydroxyvitamin D, *HbA1C* hemoglobin A1c, *HDL* high-density lipoprotein, *LDL* low-density lipoprotein, *eGFR* estimated glomerular filtration rate, *Ca* calcium, *VAS* visual analog scale, *EQ-5D* The EuroQol 5 dimensions, *Fe* iron, *Mg* magnesium

Multivariate Poisson regression analysis showed that the allocation of osteoporosis treatment (Bisphosphonate monotherapy: RR 1.42, 95% CI 1.05–1.92, *p* = 0.02), the number of pre-existing VFs (RR 1.17, 95% CI 1.08–1.25, *p* < 0.01), the grade of pre-existing VFs (e.g., Grade 1 vs. None: RR 8.08, 95% CI 3.80–17.20, *p* < 0.01), prior treatment for osteoporosis (RR 0.74, 95% CI 0.55–0.99, *p* = 0.04), serum triglyceride level (per 10 mg/dL: RR 0.94, 95% CI 0.91–0.97, *p* < 0.01), and low back pain VAS score (at rest, per 10 points: RR 1.09, 95% CI 1.04–1.15, *p* < 0.01) were independent factors (Table 4). In the block-wise analyses, the PF model identified both the TUG test (RR 1.10, 95% CI 1.04–1.15, *p* < 0.01) and nursing care level 1 (RR 1.82, 95% CI 1.06–3.13, *p* = 0.03) as predictors of incident vertebral fractures (Table 5). In contrast, in the BTM and NS models, none of the additional variables were associated with fracture risk (Tables 6 and 7). Across all block-augmented models, the direction and significance of the core predictors (treatment allocation, prevalent vertebral fractures, back pain VAS, and triglycerides) remained consistent.

**Table 4:** Multivariable analysis of associations between morphometric vertebral fractures and risk factors selected by a stepwise procedure

	Rate ratio	95% CI		*p*
Allocation of osteoporosis treatment Bisphosphonate monotherapy (Reference = Teriparatide → Alendronate sequential therapy)	1.42	1.05	1.92	0.02*
Count of pre-existing vertebral fractures	1.17	1.08	1.25	< 0.01*
Maximum grade of pre-existing vertebral fractures				
None	Reference			
Grade 1	8.08	3.80	17.20	< 0.01*
Grade 2	7.09	3.44	14.63	< 0.01*
Grade 3	4.08	1.94	8.57	< 0.01*
Prior treatment	0.74	0.55	0.99	0.04*
Triglycerides (per 10 mg/dL)	0.94	0.91	0.97	< 0.01*
VAS at rest (per 10 points from 0 to 100 points)	1.09	1.04	1.15	< 0.01*

The Akaike Information Criterion (AIC) and Bayesian Information Criterion (BIC) were used to assess model fit, with lower values indicating a better model. The values for the final model were AIC = 881.2 and BIC = 923.1 *p < 0.05*CI* confidence interval, *VAS* visual analog scale

**Table 5:** Multivariable Poisson regression for incident vertebral fractures: Block model including physical function variables

	Rate ratio	95% CI		*p*
Allocation of osteoporosis treatment Bisphosphonate monotherapy (Reference = Teriparatide → Alendronate sequential therapy)	1.40	1.03	1.89	0.03*
Count of pre-existing vertebral fractures	1.16	1.08	1.25	< 0.01*
Maximum grade of pre-existing vertebral fractures				
None	Reference			
Grade 1	7.75	3.62	16.57	< 0.01*
Grade 2	6.95	3.37	14.33	< 0.01*
Grade 3	3.89	1.85	8.18	< 0.01*
Prior treatment	0.74	0.55	1.00	0.05
Triglycerides (per 10 mg/dL)	0.99	0.99	1.00	< 0.01*
VAS at rest (per 10 points from 0 to 100 points)	1.10	1.04	1.15	< 0.01*
Timed-up-and-go test (seconds)	1.10	1.04	1.15	< 0.01*
Nursing level				
Support not required	1.01	1.00	1.02	0.19
Support required	1.02	0.66	1.58	0.93
Level 1	1.82	1.06	3.13	0.03*
Level 2	0.36	0.09	1.46	0.15
Level 3	0.64	0.15	2.63	0.53
Level 4 or 5	1.03	0.25	4.29	0.97

The Akaike Information Criterion (AIC) and Bayesian Information Criterion (BIC) were used to assess model fit, with lower values indicating a better model. For this block model, the values were AIC = 883.8 and BIC = 953.6 *p < 0.05*CI* confidence interval, *VAS* visual analog scale

**Table 6:** Multivariable Poisson regression for incident vertebral fractures: Block model including bone turnover and bone quality markers

	Rate ratio	95% CI		*p*
Allocation of osteoporosis treatment Bisphosphonate monotherapy (Reference = Teriparatide → Alendronate sequential therapy)	1.46	1.08	1.98	0.02
Count of pre-existing vertebral fractures	1.16	1.08	1.25	< 0.01*
Maximum grade of pre-existing vertebral fractures				
None	Reference			
Grade 1	8.06	3.78	17.16	< 0.01*
Grade 2	6.83	3.31	14.13	< 0.01*
Grade 3	3.95	1.88	8.32	< 0.01*
Prior treatment	0.79	0.57	1.10	0.16
Triglycerides (per 10 mg/dL)	0.99	0.99	1.00	< 0.01*
VAS at rest (per 10 points from 0 to 100 points)	1.08	1.03	1.14	< 0.01*
TRACP-5b (mU/dL)	1.02	0.96	1.10	0.50
Pentosidine (pmol/mL)	1.00	1.00	1.01	0.07
BMD at L2-L4 (*T*-score)	1.01	0.88	1.15	0.91

The Akaike Information Criterion (AIC) and Bayesian Information Criterion (BIC) were used to assess model fit, with lower values indicating a better model. For this block model, the values were AIC = 882.2 and BIC = 938.1 *p < 0.05*CI* confidence interval, *VAS* visual analog scale, *TRACP-5b* tartrate-resistant acid phosphatase-5b, *BMD* bone mineral density

**Table 7:** Multivariable Poisson regression for incident vertebral fractures: Block model including nutritional and social factors

	Rate ratio	95% CI		*p*
Allocation of osteoporosis treatment Bisphosphonate monotherapy (Reference = Teriparatide → Alendronate sequential therapy)	1.43	1.05	1.94	0.02
Count of pre-existing vertebral fractures	1.15	1.07	1.24	< 0.01*
Maximum grade of pre-existing vertebral fractures				
None	Reference			
Grade 1	8.28	3.89	17.63	< 0.01*
Grade 2	7.01	3.39	14.51	< 0.01*
Grade 3	4.02	1.91	8.46	< 0.01*
Prior treatment	0.79	0.58	1.06	0.12
Triglycerides (per 10 mg/dL)	0.99	0.99	1.00	< 0.01*
VAS at rest (per 10 points from 0 to 100 points)	1.08	1.03	1.14	< 0.01*
Albumin (g/dL)	0.67	0.45	0.99	0.05
Alcohol intake (g)	1.00	1.00	1.01	0.05
Number of teeth present	0.99	0.98	1.01	0.41
HbA1C (%)	0.87	0.66	1.15	0.33

The Akaike Information Criterion (AIC) and Bayesian Information Criterion (BIC) were used to assess model fit, with lower values indicating a better model. For this block model, the values were AIC = 880.2 and BIC = 940.7 *p < 0.05*CI* confidence interval, *VAS* visual analog scale

## Discussion

Univariate and multivariate analyses showed that sequential therapy with once-weekly teriparatide followed by a bisphosphonate resulted in a lower incidence of new VFs than bisphosphonate monotherapy. These results were consistent with the previous findings that once-weekly teriparatide has a stronger fracture prevention effect than bisphosphonates [19]. This finding is interesting given that increases in BMD with once-weekly teriparatide were similar to those seen with bisphosphonates [19]. Teriparatide improves bone quality by activating bone remodeling [23]. In a study comparing vertebral strength properties between patients treated with daily teriparatide and alendronate using finite element modeling of QCT scans, teriparatide affected vertebral strength by altering density distribution within the vertebrae, with teriparatide producing a fivefold increase in the strength-to-density ratio compared with bisphosphonates [24]. Once-weekly teriparatide has a moderate effect on cancellous bone, increasing cortical thickness and total bone strength to a similar extent as daily teriparatide [25]. These stronger effects of once-weekly teriparatide on bone strength compared with bisphosphonates may have led to the greater fracture prevention effect of teriparatide in the present study.

The results of the present study showed that the incidence of new VFs was higher in patients with pre-existing VFs than in those without. Furthermore, a positive association was observed between the number of existing VFs and the occurrence of new fractures. This finding aligns with previous research, which indicates that a greater number of VFs increases the likelihood of subsequent fractures [26]. The results of the present study support this conclusion, suggesting that existing VFs are indicative of an elevated fracture risk and a deterioration of spinal alignment. Therefore, preventing the initial occurrence of VFs is crucial. Interestingly, though the presence of any VF was associated with the occurrence of new VFs, the results did not show a clear increase in fracture risk based on the severity of the deformity as classified by the SQ grade. This suggests that even minor spinal deformities resulting from VFs are associated with subsequent fracture events. A population-based cohort study showed that patients with pre-existing mild VFs had a higher incidence of new VFs compared to those without any pre-existing VFs at baseline (3-year incidence: 37.3% vs. 12.2%) [7]. This suggests that even a grade 1 VF may be sufficient to alter the biomechanics of the entire spine, serving as an indicator of fragility that increases the risk of subsequent VFs. However, further investigation is necessary to determine the extent to which the severity of vertebral deformity affects the risk of future fractures.

Univariate analysis indicated that all lipid-related parameters were associated with the occurrence of new VFs. The regression coefficient suggested that hyperlipidemia is inversely associated with fracture risk. Furthermore, the triglyceride level remained a significant variable in the final multivariate model. Dyslipidemia is often associated with obesity; however, the relationship between obesity-related adipose tissue and bone metabolism is complex. On one hand, increased adipose tissue leads to higher aromatase activity and subsequent estrogen production [27], which is bone-protective. On the other hand, visceral adipose tissue, in particular, secretes pro-inflammatory cytokines that can promote bone resorption [28]. Accordingly, the relationship between dyslipidemia and osteoporotic fractures remains debated in the literature. Whereas some reports suggest that high variability in total cholesterol levels increases fracture risk, findings regarding the effect of elevated total cholesterol levels are inconsistent [29]. Similarly, there is no consensus on the associations between HDL cholesterol, LDL cholesterol, or triglyceride levels and the risk of fracture [30]. Specifically concerning VFs, high total cholesterol variability and obesity have been proposed as potential risk factors [31, 32]. Conversely, other studies have reported a protective effect of high total cholesterol levels against fracture [33, 34]. The present findings, which suggest that lower triglyceride levels could increase the risk of VFs, are consistent with the hypothesis that hyperlipidemia may be preventive. Nevertheless, this observation is preliminary and warrants further validation through dedicated prospective studies.

In the present study, the VAS score for lower back pain was independently associated with the occurrence of new VFs. This finding is consistent with previous research demonstrating that severe lower back pain correlates with a higher incidence of subsequent VFs [35]. Although the underlying mechanism remains unclear, it is hypothesized that severe pain may lead to reduced physical activity and immobilization. This state of disuse can contribute to bone demineralization, potentially creating a “domino effect” of further fractures. Based on these results, effective pain management in the acute phase of a fracture is crucial for preventing subsequent fractures. The National Institute for Health and Care Excellence guidelines for long bone fractures recommend a multimodal approach to analgesia for severe acute pain, combining medications with different mechanisms of action rather than relying on monotherapy [36]. Accordingly, clinicians should aim to thoroughly control pain in patients with acute VFs, considering a combination of acetaminophen, non-steroidal anti-inflammatory drugs, and opioids. Future randomized, controlled trials are warranted to determine which specific analgesics and combination therapies are most effective for post-VF pain.

Regarding the relationship between bone metabolic markers and VFs, the univariate regression analysis showed that TRACP-5b and pentosidine levels were significantly associated with the risk of fracture, although they were not included in the final multivariate model. A recent retrospective study investigating the incidence of fractures in osteoporotic patients with VFs found that the concentration of C-terminal telopeptide of type 1 collagen was associated with the incidence of fractures, whereas PINP was not [37]. In the present study, the bone resorption marker TRACP-5b was significantly involved in the occurrence of fractures, whereas the bone formation marker PINP was not. This suggests that, in patients with established severe osteoporosis and pre-existing VFs, ongoing high rates of bone resorption are an important pathological process that promotes persistent bone loss and increases the risk of further fractures. Though the measurement of bone formation markers may still have a role in monitoring treatment response and predicting fractures in the non-severe osteoporotic population, the measurement of bone resorption markers appears to be more informative for predicting impending re-fracture in patients with severe osteoporosis.

Our findings confirmed that treatment allocation, prevalent vertebral fractures, pain severity, and triglyceride levels were consistent predictors of new vertebral fractures. In addition to the stepwise-selected model, we performed supplementary block-wise analyses to address the concern that clinically relevant predictors might have been omitted. These analyses demonstrated that functional measures such as the TUG test and nursing care level were independently associated with incident vertebral fractures, whereas bone turnover markers (TRACP-5b, pentosidine, BMD) and nutritional/social factors (albumin, alcohol intake, number of teeth) did not reach statistical significance. Importantly, across all block-augmented models, the direction and significance of the core predictors—including treatment allocation, prevalent vertebral fractures, back pain VAS, and triglyceride levels—remained consistent. Although stepwise selection methods are often criticized for instability and risk of overfitting [35], our analyses demonstrate that the main predictors identified in the primary model were robust to alternative modeling strategies. Moreover, the consistency of key predictors across models with favorable AIC and BIC values further supports the robustness of the findings. Taken together, these findings suggest that both pharmacologic treatment and baseline skeletal damage remain the strongest determinants of new vertebral fractures in this population, while functional decline adds complementary prognostic value. Future studies with larger and more diverse cohorts are warranted to validate the role of physical function measures and to further explore nutritional and metabolic factors in fracture prediction.

The limitation of this study is that it was a post hoc analysis and not a prospective study. Therefore, there could be factors that contribute to the occurrence of fractures that were not addressed in this study. In addition, the participants were elderly women, and it may be difficult to directly apply the results of this study to men or younger patient groups. Finally, the management of new vertebral fractures was not standardized; the varied use of interventions such as brace therapy and analgesics for fractures that occurred during the observation period could have potentially affected the fracture incidence rate.

## Conclusion

Preventing VFs in patients with severe osteoporosis requires a comprehensive risk assessment that incorporates factors beyond BMD. The present findings establish that the specifics of osteoporosis treatment, the burden of existing fractures, the intensity of back pain, and even low triglyceride levels are crucial, independent predictors. Therefore, clinical strategies to reduce future fracture risk in high-risk patients should include enhancing osteoporosis therapy (e.g., with teriparatide), maintaining adequate triglyceride levels, and using multimodal analgesia for pain control. Furthermore, future studies are warranted to determine the optimal sequential or combination regimens using other potent anabolic agents, such as romosozumab or abaloparatide, with various anti-resorptive agents (e.g., zoledronic acid, denosumab, or oral bisphosphonates) to maximize fracture prevention in this high-risk population.

## Data Availability

The data that support the findings of this study are available from the corresponding author upon reasonable request.
